# Ethical Issues in Pharmacologic Research in Women Undergoing Pregnancy Termination: A Systemic Review and Survey of Researchers

**DOI:** 10.1155/2012/724591

**Published:** 2011-11-30

**Authors:** Christelle Gedeon, Alejandro A. Nava-Ocampo, Gideon Koren

**Affiliations:** ^1^Motherisk Program, Division of Clinical Pharmacology and Toxicology, The Hospital for Sick Children, Toronto, ON, Canada M5G 1X8; ^2^Department of Pharmacology, Faculty of Medicine, University of Toronto, Toronto, ON, Canada M5S 1A1

## Abstract

*Objective*. To evaluate the ethics of performing research in the field of maternal-fetal medicine involving women undergoing pregnancy termination. *Methods*. We identified published pharmacological studies performed during elective pregnancy termination. In addition, a questionnaire was administered to investigate whether this research would be acceptable to professionals performing research in the field of maternal-fetal pharmacology. *Results*. The majority of participants believe that this form of research is necessary to furthering our understanding of drug use in pregnancy. Twenty studies were identified in women undergoing a pregnancy termination where exogenous drug was administered and drug measurement conducted during an abortion. The majority of studies were completed by international groups and not in North America or Western Europe. *Conclusions*. While a majority of respondents to the survey felt that, although research in women undergoing a pregnancy termination is ethically acceptable, 40% stated that it is not likely to be approved by institutional review boards of most North American medical institutions.

## 1. Introduction

Approximately 50% of pregnancies are unplanned [1] often making drug exposure in the first trimester unintentional. However, once a pregnant woman becomes aware of her pregnancy, a series of ethical and legal dilemmas surface. In many cases, the lack of information on safety of specific drugs in pregnancy is a problem when deciding on drug alternatives to treat or modify therapy in pregnant patients.

The information regarding teratogenicity obtained in experimental animal models is usually difficult to extrapolate to clinical decision. Most drugs that have exhibited teratogenic effects in humans have also been proven to be teratogenic in animal models [2], but not all drugs with proven teratogenicity in animal models have been found to be human teratogens [2].

*Ex vivo*, human placental studies have proven to be invaluable tools to answer questions regarding the trans-placental transfer of drugs [2]. However, since the *ex vivo* model uses placentas obtained at delivery, it often cannot necessarily provide useful information with regards to the first or second trimesters of pregnancy. Clinical experience is therefore critical to understand the complexity of drug behavior in pregnancy.

Because ethical challenges have made it difficult to study drug safety in pregnant women, an intriguing line of clinical research has been conducted over the last 4 decades in women undergoing pregnancy termination. This research, conducted worldwide, has focused on the study of the aborted fetus with regards to drug absorption and disposition. The objective of the present study was to systematically identify studies performed in women undergoing pregnancy termination where biological samples were obtained in order to investigate transplacental drug transfer. Subsequently, we aimed to evaluate the attitudes of scientists and physicians practicing in the field of maternal-fetal medicine with regards to studies involving women undergoing pregnancy termination.

## 2. Methods

### 2.1. Systematic Review

Search was performed in Pubmed-Medline and M-Base for studies in women undergoing voluntary pregnancy termination who received a drug not aimed for their health. Search limits included a date span from January 1964 to 2006 and only English articles were included.

### 2.2. Survey

To investigate the attitudes of health professionals on ethical aspects of research in pregnant women prior, during and after abortion, we designed a questionnaire consisting of 2 hypothetical case studies. The first addressed the acceptability sampling of amniotic protein levels during an abortion, while a second case described a study of the administration of the antiretroviral drug, zidovudine, to a woman undergoing an abortion infected with HIV. The questionnaire collected data on gender and country of origin of the participants, as well as an open comments section to express personal views on the acceptability of the presented studies was offered. Participants were asked if such procedures would be acceptable in the different trimesters of pregnancy, single versus multiple dose of drug administration to the mother, and whether their institutional ethics board would approve such research protocols.

Questionnaires were distributed during two maternal-fetal pharmacology workshops, in Denver, Colo, USA, and Toronto, ON, USA. Participants included both basic and clinical scientists performing research in the area of maternal-fetal medicine and toxicology. Participation was voluntary and this study received ethical approval by The Hospital for Sick Children's research ethics board.

## 3. Results

### 3.1. Systematic Review

Twenty studies were identified, where a drug was intentionally administered to a woman undergoing an abortion. One forensic report of a woman exposed to heroin in pregnancy was also included (Table 1). Of them, 6 (28.6%) were performed in the 70's, 4 (19%) were performed in the 80's, 6 (28.6%) were performed in the 90's, and 5 (23.8%) in the 2000's. Only four (19%) studies were reported from North America, and all were reported between 1977 and 1983 whereas 17 (81%) studies were performed in other parts of the world. The studied exogenous compounds varied from ethanol and glucose to therapeutic drugs. The route of administration to the mother was mainly oral. Nine (42.9%) of the studies evaluated multiple-dose administration and 8 (38.1%) evaluated a single dose of the exogenous compound. Of the 21 studies, 5 (23.8%) included women in the first trimester, 7 (33.3%) included in the second trimester, 8 (38.1%) included women in the 1st-2nd trimesters, and only one study reported on women in the last trimester of pregnancy.

Excluding the forensic case, 6 (30%) studies did not declare ethics approval from their institutions and 3 (15%) studies did not mention obtaining informed consent from the participants.

### 3.2. Survey

Fifty participants responded to the administered questions (26 women and 24 men). In such a protocol with 56.0% preferring to conduct the study in the first trimester, 21% in the second trimester, and 27.0% at anytime during pregnancy. In response to the scenario of exogenous drug administration, 34 (69%) respondents considered the study design as acceptable. Of them, men and women did not differ in their responses. Participants preferred to conduct the study in 1st-trimester abortions (42%), while the options of second trimester (17%) or anytime during pregnancy (31%) were less favored. With regards to dosing, 55% of participants preferred a single dose administration.

Of interest, 39.0% of participants, while considering the proposed research to be useful, believed that their institutional review boards were likely not to approve the design. Another 39.0% of participants (50.0% men and 30.0% women) considered that this type of research would be “too controversial.” Finally, 20.0% of participants, all women, indicated that they would not perform this research because of fear of criticism leaving the remaining 11% of participants, who would not perform this research because of personal reasons.

## 4. Discussion

In this study, we sought to investigate whether clinical trials in women undergoing pregnancy termination are ethically viable for the assessment of safety and transplacental drug transport in pregnancy. This is the first study to systematically review this topic and collect data on the attitudes of researchers regarding the ethical standards necessary to perform pharmacokinetic trials in women undergoing pregnancy termination.

The systematic review identified 20 studies of fetal drug distribution in women undergoing pregnancy termination performed since the 1970's. Studies of exogenous compounds were performed largely outside of North America between the 80's and 90's. The lack of North American abortive research may be due to dissimilar attitudes regarding abortion as compared to Europe or Asia. For example, in Sweden, legal abortion is a recurrent part of a gynecologists' work. A survey of Swedish gynecologist found that almost all believed that gynecologists should be involved in abortion care and half were opposed to the privilege of refusing to work with termination of pregnancy [22]. Alternatively, it has been suggested that the lack of North American studies in the abortus population may be due to the ethical standards in north American institutions becoming more stringent with the advent of fetal tissue research and *in utero* fetal surgery.

Of importance, 30% of those studies, despite the utmost sensitivity of the clinical situation, do not mention approval by the local ethics committee. Such approval is a legal and ethical prerequisite in all countries where these studies had been performed.

Our survey of the attitudes of North American researchers towards research in women undergoing abortion suggests that the majority of researchers in maternal-fetal medicine would respond positively to performing studies in women undergoing a pregnancy termination. Their opinions were not different from those of surveyed women [23]. According to Anderson et al., 94% of women feel that fetal tissue research was justifiable and 84% say they would allow this sort of research to be done on their own fetus [23]. In fact, this same study [23] found that, while few women felt that research on a live fetus was justifiable, significantly more women (68%) about to undergo termination of pregnancy found this idea acceptable and more than half of them would have permitted research to be carried out on their own live fetus [23]. Our present survey results are in agreement with those of Anderson et al. [23]. Both women about to undergo an abortion and our surveyed health professionals were in agreement with regards to the value of studies and information that may results from studies in the abortive population.

Overwhelmingly, the opinion of our surveyed professionals was that this type of research was useful but likely not to be approved by their respective institutions. The common fear was that such a study design would be too controversial. However, pregnancy is a dynamic state that can only be compared to itself. Without the participation of pregnant women themselves, safe and effective use of drugs during this critical period in a women's life will in no way become a reality. However, the abortive population presents itself as a relevant pharmacological comparison, to be used as an investigational population for drugs in pregnancy without the risk of any fetal adverse consequences.

The open comments section of the survey was the most informative with regards to justifications against research in women undergoing pregnancy terminations. The most pertinent reason was with regards to the quality of consent that can practically be achieved in a women undergoing pregnancy termination.

Since the Nuremberg Code, it has been widely established that ethical consent cannot be obtained from a patient who is under coercion, threat, or duress [24]. With regards to abortion, it could be argued that the consent process is in itself intrinsically coercive due to the emotional nature of the situation [25]. It has been suggested that women would be more likely to consent to participate in research as a way to alleviate their feelings of guilt surrounding the abortion [23]. Currently, in order to improve the quality of consent in the abortive population, the objective has been to achieve a practical separation of the abortion and the subsequent use of human fetal tissue [23]. The same should apply to a woman's decision to enroll in a pharmacokinetic study. In fact, Bopp Jr. observed that most women were ambivalent about abortion, with 5% changing their minds after making the abortion appointment [26]. 

As such, the Human Fetal Tissue Transplantation Research (HFTTR) panel recommended that a woman should not be asked for her consent to participate in a study until she has decided to obtain an abortion [8]. If such research is to happen, the women should be recruited by a recruiter not affiliated with the abortion clinic, capable of assessing the women's capacity to consent, considering her level of education, language comprehension, and cultural adaptation to an authority figure (i.e., doctor) making the request. The hope is that an independent recruiter will circumvent the risk of coercion by separating the “source” (the patient providing the samples) and the “user” (the researcher employing the samples) [27].

In addition, the consent process should be value neutral, neither approving nor disapproving of the practice of abortion. Thus, the consent process should be nondirective and noncoercive [27]. Chervenak et al. [27] have suggested that words such as “treatment” and “therapy” should not be used by the investigator to describe the intervention. Words such as “mother, father, and baby” should not be used because these suggest moral relationships. Instead, it is preferable to use words such as “pregnant woman” and “abortus.” The consent form should also contain explicit details about the nature of the procedure, including the risks regarding future pregnancy and postpartum management following the study. A related recommendation, then, is that researchers should routinely test the participating woman's understanding of risk for the individual study, a practice endorsed by many and practiced by few. Autonomous decision making is a core requirement of ethical research. Vigilance about minimizing and managing study risks is likely the best protection volunteers can have [28].

There were several limitations to this study. Firstly, there were 50 respondents recruited predominantly at 2 academic meetings in North America. This might have created a bias as those likely not to participate at meetings may have different views and may less likely approve such a protocol. Secondly, interviewing a larger, more heterogeneous study population will help to confirm or reject our findings. Finally, by design, this was not a prevalence study. Targeting enrollment in this way did not allow examination of questions related to prevalence.

## 5. Conclusions

We have identified published studies investigating the trans-placental pharmacology in women undergoing abortion and have discussed some of their ethical implications. Progress in pregnancy drug use, design, and testing can only be made possible through the participation of pregnant women themselves. It is of interest that very few of them were conducted in North America in the last decade, while this type of research has been practiced in different parts of the world. The ability to test an abortus and fetal tissue is a relatively novel strategy, accepted by the majority of surveyed women and scientists in the field of maternal fetal pharmacology. It is our contention that the North American discomfort with the use of the abortive population, ingrained in our scientific institutions, will hinder our ability to improve drug safety and treatment in maternal-fetal pharmacology. The potential information gained from conducting such studies may be important to bridging the knowledge gap in terms of drug behavior in pregnant women and providing the necessary information to alleviate fears of prescribing drugs in pregnancy. In this way, the experience of abortus research has the potential to be transformed into a life-saving opportunity for future fetuses. Yet the extreme vulnerability and sensitivity of the woman during this time dictates that continued discussion of the risks, benefits, and quality of consent continue to take place.

Much larger debate should ensue to try to resolve these issues.

##  Conflict of Interests

There are no conflict of interests of any of the authors in the preparation of this paper.

##  Disclosure

G. Koren has the right to grant on behalf of all authors and does grant on behalf of all authors, an exclusive license (or nonexclusive for government employees) on a worldwide basis to the BMJ Publishing Group Ltd and its licenses to permit this paper (if accepted) to be published in JME and any other BMJPGL products to exploit all subsidiary rights, as set out in our license (http:/group.bmj.com/products/journals/instructions-for-authors/licence-forms).

## Figures and Tables

**Table 1:** Studies where exogenous drug was administered and sampled from women and their abortus prior to pregnancy termination.

Reference	Year	Country	Xenobiotic
[3]	2005	Hong Kong	Rosiglitazone
[4]	2002	Hong Kong	Naproxen
[5]	2002	Israel	(15S)-15 Methyl PG F2alpha
[6]	2002	Belgium ans UK and Madrid	Cefoxitin, moxalactam and ceftazidime
[7]	1999	Belgium and UK	Glucose
[8]	1999	France	Diclofenac
[9]	1998	Belgium and UK	Fentanyl
[10]	1996	Belgium and UK	Diazepam
[11]	1993	Belgium and UK	Fentanyl
[12]	1993	Germany	Thyroxine
[13]	1983	Canada	Ethanol
[14]	1980	Finland	Oxazepam
[15]	1978	Czech	Sodium salicylate
[16]	1977	USA	Cefazolin
[17]	1977	USA	Amikacin
[18]	1997	UK	Inulin
[19]	1977	USA	Tobramycin
[20]	1999	USA	Heroin
[21]	1975	Singapore	Thiamphenicol

GA: gestational age; IRB: ethics approval obtained; Consent: clearly declared consent obtained; Mat: maternal blood samples; Fetal: fetal blood samples obtained; Amniotic: amniotic/celomic samples obtained; FT: fetal tissue collected and sampled.

## References

[1] Koren G (2001). *Maternal Fetal Toxicology. A Clinician’s Guide*.

[2] Bourget P, Roulot C, Fernandez H (1995). Models for placental transfer studies of drugs. *Clinical Pharmacokinetics*.

[3] Chan LYS, Yeung JHK, Lau TK (2005). Placental transfer of rosiglitazone in the first trimester of human pregnancy. *Fertility and Sterility*.

[4] Siu SSN, Yeung JHK, Lau TK (2002). An in-vivo study on placental transfer of naproxen in early human pregnancy. *Human Reproduction*.

[5] Weinstein L, Droegemueller W, Cornette J (1979). (15S)-15 methyl prostaglandin F2*α* levels in amniotic fluid and blood in second trimester abortions. *Southern Medical Journal*.

[6] Giamarellou H, Gazis J, Petrikkos G (1983). A study of cefoxitin, moxalactam, and ceftazidime kinetics in pregnancy. *American Journal of Obstetrics and Gynecology*.

[7] Weissman A, Lowenstein L, Drugan A, Zimmer EZ (2003). Effect of the 100-g oral glucose tolerance test on fetal acid-base balance. *Prenatal Diagnosis*.

[8] Siu SSN, Yeung JHK, Lau TK (2000). A study on placental transfer of diclofenac in first trimester of human pregnancy. *Human Reproduction*.

[9] Shannon C, Jauniaux E, Gulbis B, Thiry P, Sitham M, Bromley L (1998). Placental transfer of fentanyl in early human pregnancy. *Human Reproduction*.

[10] Jauniaux E, Jurkovic D, Lees C, Campbell S, Gulbis B (1996). In-vivo study of diazepam transfer across the first trimester human placenta. *Human Reproduction*.

[11] Cooper J, Jauniaux E, Gulbis B, Quick D, Bromley L (1999). Placental transfer of fentanyl in early human pregnancy and its detection in fetal brain. *British Journal of Anaesthesia*.

[12] Marqusee E, Hill JA, Mandel SJ (1997). Thyroiditis after pregnancy loss. *Journal of Clinical Endocrinology and Metabolism*.

[13] Brien JF, Loomis CW, Tranmer J, McGrath M (1983). Disposition of ethanol in human maternal venous blood and amniotic fluid. *American Journal of Obstetrics and Gynecology*.

[14] Kangas L, Erkkola R, Kanto J, Eronen M (1980). Transfer of free and conjugated oxazepam across the human placenta. *European Journal of Clinical Pharmacology*.

[15] Elis J, Sechserova M, Stribrny J, Drabkova J (1978). The distribution of sodium salicylate in the human fetus. *International Journal of Clinical Pharmacology Therapy and Toxicology*.

[16] Bernard B, Barton L, Abate M, Ballard C (1977). Maternal fetal transfer of cefazolin in the first twenty weeks of pregnancy. *Journal of Infectious Diseases*.

[17] Bernard B, Abate M, Thielin PF (1977). Maternal fetal pharmacological activity of amikacin. *Journal of Infectious Diseases*.

[18] Jauniaux E, Lees C, Jurkovic D, Campbell S, Gulbis B (1997). Transfer of inulin across the first-trimester human placenta. *American Journal of Obstetrics and Gynecology*.

[19] Bernard B, Garcia Cazares SJ, Ballard CA (1977). Tobramycin: maternal fetal pharmacology. *Antimicrobial Agents and Chemotherapy*.

[20] Pötsch L, Skopp G, Emmerich P, Becker J, Ogbuhui S (1999). Report on intrauterine drag exposure during second trimester of pregnancy in a heroin-associated death. *Therapeutic Drug Monitoring*.

[21] Nau H, Welsch F, Ulbrich B (1981). Thiamphenicol during the first trimester of human pregnancy: placental transfer *in vivo*, placental uptake *in vitro*, and inhibition of mitochondrial function. *Toxicology and Applied Pharmacology*.

[22] Hammarstedt M, Lalos A, Wulff M (2006). A population-based study of Swedish gynecologists' experiences of working in abortion care. *Acta Obstetricia et Gynecologica Scandinavica*.

[23] Anderson F, Glasier A, Ross J, Baird DT (1994). Attitudes of women to fetal tissue research. *Journal of Medical Ethics*.

[24] Herbert P (1995). *Doing Right: A Practical Guide to Ethics for Physicians and Medical Trainees*.

[25] Reardon L (1999). The ethics of fetal tissue transplant research: a review. *The Linacre Quarterly*.

[26] Bopp J, Cataldo P, Moraczewski A (1994). Fetal tissue transplantation and moral complicity with induced abortion. *The Fetal Tissue Issue: Medical and Ethical Aspects*.

[27] Chervenak FA, McCullough LB, Birnbach DJ (2004). Ethical issues in fetal surgery research. *Best Practice and Research: Clinical Anaesthesiology*.

[28] Kass NE, Myers R, Fuchs EJ, Carson KA, Flexner C (2007). Balancing justice and autonomy in clinical research with healthy volunteers. *Clinical Pharmacology and Therapeutics*.

